# Emotions spread like contagious diseases

**DOI:** 10.3389/fpsyg.2025.1493512

**Published:** 2025-04-09

**Authors:** Hao Liu, Dong Zhang, Yaning Zhu, Hongwen Ma, Hongling Xiao

**Affiliations:** ^1^Nursing Department, Tianjin Union Medical Center, The First Affiliated Hospital of Nankai University, Tianjin, China; ^2^Department of Urology, Tianjin Union Medical Center, The First Affiliated Hospital of Nankai University, Tianjin, China; ^3^School of Nursing, Zhejiang Chinese Medicine University, Zhejiang, China

**Keywords:** emotional contagion, human, animals, behavior mimicry, online social network, model

## Abstract

Emotional contagion, that is, the spontaneous synchronization of emotions among individuals, is the basic mechanism of social cohesion and survival of different species. Emotional contagion can be observed in humans and many animals, and it has become an effective means to protect individuals from danger. The study of emotional contagion in different animals is of evolutionary significance, and in human society, emotional contagion has an important impact on mental health and group behavior. However, the existing research still has obvious shortcomings in the similarities and differences of cross-species emotional contagion, the communication dynamics in virtual space and the infectious effect of positive emotions. This paper reviews the mechanisms of emotional contagion in different species, such as rodents, nonhuman primates, dogs, crows and zebrafish, highlighting evolutionary conservatism and species-specific adaptation, and analyzes the role of human children's behavioral mimicry in its early development. Furthermore, we extend the discussion of emotional contagion to virtual social networks, revealing the unique communication mechanism in online environment. In addition, by combing the sociological model and the emerging neurocognitive model, the complex process of emotional contagion can be explained more comprehensively. Through multidisciplinary perspective, it provides systematic theoretical and empirical support for us to understand emotional contagion.

## 1 Introduction

We can copy the emotions of others and produce corresponding changes in behavior and neural signals, a special ability known as emotional contagion. Hatfield et al. define emotional contagion as “Observer's tendency to automatically imitate and synchronize the facial expressions, voice, posture and movements of the demonstrator, and thus to emotionally converge” (Hatfield et al., 1994). Emotional contagion differs from mimicry in that the latter implies copying the demonstrator's actions with only behavioral changes and no emotional overtones, whereas the former takes into account the matching of actions and emotions. This means that emotional contagion should satisfy both behavioral mimicry and emotional change, and that behavioral change alone does not prove the emergence of emotional contagion. For example, contagious yawning is still considered to be an imitative behavior rather than a higher dimensional emotional contagion or empathy phenomenon (Gallup, 2021). The process of emotional contagion should therefore follow a two-step model: First, the observer recognizes the change in the demonstrator's behavior, and second, the observer imitates these behaviors and has the same emotional experience (Dezecache et al., 2015). It is important to note that the process of emotional contagion is not necessarily the observer's awareness of another person's emotions, but more of a transfer of emotions (Adriaense et al., 2019). Emotional contagion becomes an effective self-protection mechanism when fearful or painful behavior is observed in similar species. When the demonstrator perceives danger or reacts to a stimulus, the observer will react in the same way to protect themselves by witnessing the demonstrator's behavior, even if they do not perceive the presence of danger. Similarly, positive emotions are widely transmitted in groups and create strong bonds between individuals in a population. Although the study of positive emotional contagion still represents a very small fraction of research, it is of extraordinary research significance. In addition, emotional contagion is an important building block for the production of empathy, and the emergence of the capacity for emotional contagion is a harbinger of an animal's evolution to a higher emotional dimension. As a common social psychological phenomenon, emotional contagion not only affects human behavior and decision-making, but also plays an important role in the animal kingdom. From the perspective of evolution, emotional contagion may contribute to information transmission and group cooperation among individuals, thus improving the success rate of survival.

The process of emotional contagion involves many aspects within the fields of sociology, psychology, and biology, and in-depth research on emotional contagion requires multidisciplinary collaboration and the use of research tools and theoretical frameworks from different fields to rationalize the process of emotional contagion. This paper aims to illustrate the prevalence of emotional contagion in both animal and human populations, beginning with a review of research on emotional contagion among animals from the perspective of animals of different species and a preliminary introduction to cross-species emotional contagion. Afterwards, the underlying logic of emotional contagion, mimicry, is explored from the perspective of the human toddler, and emotional contagion in virtual and physical cyberspace is introduced. In human society, the scope of research on emotional contagion is no longer limited to real physical space, but also exists in virtual online communities, and emotional contagion in virtual space may be more influential. Because the social scope of an individual in the real space may be limited to his family, friends or colleagues, while in the virtual network community, the obstacle of the real distance is lifted, and the emotionally charged words, pictures or videos will be spread to more individuals and to a greater distance through the network carrier.

Finally, we also provide examples of classic and innovative theoretical models of emotional contagion, including sociological models developed from epidemiology and more recent neuroscientific models, etc. These models of emotional contagion have been used to control the spread of emotions in populations and to decipher cognitive-neurological mechanisms underlying the process of emotional contagion in human beings, but few researchers have provided comprehensive summaries of them, including the strengths and weaknesses of these theoretical models. In summary, in view of the multidisciplinary nature of this topic, it is necessary to provide a comprehensive overview to connect different fields such as sociology, psychology and neuroscience. This manuscript will construct a narrative by clearly defining the goals and findings of each part, and discuss in detail the manifestations and mechanisms of emotional contagion in different animals and humans through systematic narration and multidisciplinary perspectives, so as to provide comprehensive theoretical and empirical support for understanding this phenomenon.

## 2 Cross-species study on emotional contagion

This section will introduce in detail the relationship between mimicry behavior and emotional contagion in different animals. First of all, we will start with the study of rodents, then discuss similar studies of other species such as non-human primates, and finally summarize the importance of these studies in understanding the mechanism of emotional contagion.

### 2.1 Rodent

Rodents are the most studied animal vector for the phenomenon of emotional contagion, and most of the salient contributions to emotional contagion have been obtained from rodents. Studies on the subject date back as far as 1939 (Anderson, 1939), Until recently, the emergence of a Meta-analysis spanning 80 years and encompassing 124 studies summarized and analyzed the research on rodents within the field of emotional contagion, further clarifying the rodent model as an ideal animal model for conducting emotional contagion research. Similar to humans, rodents are highly social, and the effect of emotional contagion is even more pronounced as herd animals, features of rodent socialization may lead to large-scale emotional contagion (Nakahashi and Ohtsuki, 2015). Reviewing the previous literature, fear or anxiety remains the most frequent negative emotion in rodent emotional contagion studies, followed by pain (Hernandez-Lallement et al., 2022). In order to observe the process of emotional contagion in rodents (Church, 1959). In order to observe the process of emotional contagion in rodents, Church (1959) designed a classic emotional contagion experiment that is still worth exploring today; when one rat obtained food by pressing a lever, the other rat was simultaneously subjected to an electroshock stimulus, and when the former observed its partner's distressing emotion brought about by the electroshock, it ceased its lever-pressing behavior. This classic experiment not only demonstrated the process of emotional contagion between conspecifics, but has since led researchers to delve deeper into the question of empathy between animals of the same species. Another study placed demonstration mice in cages with flies to bite while observation mice witnessed the entire process. On day 2, when the observation mice were placed in cages that also contained flies, the mice showed painful behavior and enhanced analgesic responses even though the flies had lost their biting function (Kavaliers et al., 2001). Most researchers now use electric shocks to the soles of the demonstrator's feet, so that the observer witnesses the stimulus he or she is experiencing, and emotional contagion occurs through painful grunts, injurious movements, and so on. When witnessing the demonstrator mice writhing their bodies to minimize pain, the observer mice make the same writhing movements (Langford et al., 2006); and when the demonstration mice showed prolonged distress, the observation mice also began to show social fear behaviors such as running away (Pisansky et al., 2017). In addition, by observing only the painful behavior of mice with inflammatory pain, normal mice also showed nociceptive hypersensitivity (Smith et al., 2016). This suggests that mice may recognize pain in their own kind by sight, sound, and odor, and engage in avoidance or freezing behaviors in response to fear by means of emotional contagion, with freezing behavior being the most common rodent fear appraisal criterion (Pisansky et al., 2017). In wild environments, the emotional contagion of pain and fear can protect individuals from predators and thus perpetuate the survival of the race. In highly socialized animals, the level of emotional contagion of fear is also related to the size of the animal's population (Nakahashi and Ohtsuki, 2018). The smaller the size of an animal population, the higher the susceptibility of its fear to emotional contagion and the more likely it is to cause mass panic in the group; whereas individuals in large populations face fewer threats from predators, are better adapted to their environment, and their level of fear contagion is lower.

Stress is one of the common negative emotions in social environments, and prolonged exposure to stressful environments can be both psychologically and physiologically damaging. However, even in the absence of direct contact with the stressor, there is contagion of stress during social interactions, which is referred to as vicarious social failure and has been extensively studied in rodents, mainly rats and mice (Carnevali et al., 2020). Depression is a direct consequence of prolonged exposure to stress, and severe depression not only leads to impaired social interactions and impaired empathic responses, but prolonged exposure to depressed individuals can also be emotionally contagious to their peers, interfering with normal emotional experiences (Qu et al., 2023). Depressed rats were housed with two healthy counterparts, and all three developed depressive symptoms after 5 weeks (Boyko et al., 2015). This study provides strong evidence of the value of the emotional contagion theory within the field of psychiatric disorders, particularly the contagion of negative emotions; even without witnessing the exposure of a kindred spirit, healthy individuals can be contagious with negative emotions due to prolonged residence with an exposed kindred spirit, and some complex psychiatric disorders, such as anxiety, can be transmitted to a kindred spirit in this way as well (Meade et al., 2021).

Relevant studies on emotional contagion in rodents have been detailed in manuscripts by other researchers (Perez-Manrique and Gomila, 2022), In conclusion, the present research has found that a variety of emotions can be transmitted among rodents, such as fear, depression, and anxiety; and factors affecting the transmission of emotions in rodents, such as animal strain, previous exposure to stimuli, and familiarity with peers, have been verified, but only a few researchers have investigated the process of the transmission of positive emotions (Saito et al., 2016), and the attention of the present research is still confined to the process of the transmission of negative emotions.

### 2.2 Non-human primate

Recognizing and responding appropriately to the emotions of one's peers is essential for humans, and is considered to be an ability of all socialized animals (Ferretti and Papaleo, 2019), and this unique ability to unconsciously mimic the emotions and behaviors of others serves as “social glue” (Hale and Hamilton, 2016), and such benign social interactions increase pro-social behaviors (Wang and Hamilton, 2013). Emotional contagion is driven by behavioral mimicry, whether facial muscle mimicry or physical behavioral mimicry (Preston and de Waal, 2002). As the primate that is evolutionarily closest to humans, it has been demonstrated that it can mimic different emotions of its peers, both positive and negative, through changes in facial muscles (Parr et al., 2007), and this involuntary rapid facial mimicry (RFM) has been suggested to be a motor mirroring response, which increases emotional communication between peers (Scopa and Palagi, 2016). Primate facial expressions can currently be categorized into two types, starting with a pattern of facial muscle alterations thought to be similar to those that occur when humans produce smiles: the relaxed open mouth (ROM) or the play face (PF), a facial expression in which the mouth is held long and open but does not show all of the teeth, and human laughter is thought to have evolved from the primate PF (Fowler and Christakis, 2008); The other is full play face (FPF), in which the upper and lower teeth are exposed on top of the PF, and this bare teeth behavior usually represents submission or affiliation; therefore, these two facial expression changes are considered as one of the ways of emotional expression in primates. In a study of Bornean orangutans, it was found that most of the orangutans (*N* = 25) could mimic each other's open-mouthed expressions in a rapid period of time (<1 s), and that this phenomenon of involuntary rapid facial mimicry could be accomplished without preparation, which is related to involuntary facial mimicry for positive emotional contagion in humans (Davila et al., 2008). Lowland gorillas can observe each other's facial muscle changes during social play, and when two playmates of the same species perfectly mimic each other's facial expressions, both maintain the same expression (e.g., a big grin) for a longer period of time, whether in PF or FPF mode (Bresciani et al., 2022). And PFP is more likely to occur during intense confrontational play, which is a friendly facial expression that prevents playmates from misperceiving intense action as aggressive toward them, and that playmates can successfully recognize and respond in a friendly manner to this positive expression (Waller and Cherry, 2012), this means that positive emotions are contagious. Although rapid facial mimicry has been demonstrated in non-human primates, its influence on emotional contagion is still unclear. Future research should focus on long-term observation to verify the long-term impact of this phenomenon.

Contagious scratching and contagious yawning are two specific types of motor mimicry, and the study of these two behavioral paradigms helps us gain insight into the possible causes of behavioral mimicry in emotional contagion. Negative emotions are usually judged to be present in primates when scratching behavior is present (Palagi and Norscia, 2011), and infectious scratching has been found in studies of humans (Meeuwis et al., 2022) and primates such as rhesus monkeys (Feneran et al., 2013), Japanese macaques (Nakayama, 2004), and Bornean orangutans (Laméris et al., 2020). Interestingly, infectious scratching occurs in Mirounga leonina in addition to primates (Wojczulanis-Jakubas et al., 2019), whereas rodents, which have the highest number of occurrences in emotional contagion studies, do not have sufficient evidence to support the presence of infectious scratching (Lu et al., 2019), which is most likely the result of differences in the MNS between primates and rodents, and the fact that there are fewer studies on MNS in rodents. Whereas contagious yawning may be more helpful in our more in-depth study of emotional contagion, observing that emotionally charged facial expressions of others activate emotional contagion mechanisms, yawning, which is also a change in facial muscles but lacks emotional coloring, is similarly contagious within a group. This low-level yawning contagion helps us understand the underlying causes of emotional contagion of facial expressions, and non-human primates are excellent subjects for studying contagious yawning, not only because of their evolutionary similarities to humans, but also because of the different facial expressions they can make as well.

Contagious scratching and contagious yawning have been recognized in some primates, but more research is needed to determine if both abilities are present in all non-human primates, and more research on contagious scratching and contagious yawning in non-human primates has been combed through the manuscript of Nieuwburg et al. (2021). In summary, the current study demonstrates that low-level behavioral mimicry exists in nonhuman primate populations and that emotional contagion is equally present. These findings are of great significance for understanding the evolutionary roots of human emotional contagion. However, the results of some studies are still vague and not fully explored. For example, the discovery of positive emotional contagion in non-human primates is still limited, and further research is needed to confirm its consistency and repeatability.

### 2.3 Dog

Similar to primates, dogs have a special ability for rapid mimicry, which means that they may also possess emotional contagion. During dichotomous play interactions with conspecifics, when a playmate exhibits ROM or play bow (PBOW) behaviors, which indicate the emergence of a positive emotional state, play time between them increases by mimicking the positive behaviors of the playmate, and the level of this rapid mimicry correlates with the familiarity of the pair (Palagi et al., 2015). However, some conflicting conclusions have been drawn in studies of contagious yawning in canines. One study demonstrated that 21 out of 29 dogs would exhibit contagious yawning behavior when confronted with human yawning (Joly-Mascheroni et al., 2008). Subsequent studies have debunked this conclusion that dogs only show low levels of contagious yawning behavior, whether in the face of yawning by their own kind or by humans (Harr et al., 2009; O'Hara and Reeve, 2011). A new study then added to the initial findings that when confronted with an unfamiliar human yawning, 72% of adult dogs and 69% of puppies (4–14 months) showed contagious yawning, and 61% showed delayed yawning (e.g., yawning 5 min after the observation of a human yawning) (Madsen and Persson, 2013). The conclusion contradiction of infectious yawning in dogs may come from the difference of experimental design. The age of dogs and their emotional connection with humans may be key variables, and it is urgent to standardize the experimental paradigm to verify the reproducibility. Although it has been possible to establish that mimicry occurs among canines, their contagious mimicry of yawning in conspecifics and humans still needs to be confirmed by more research.

Dogs are one of man's reliable animal friends, and in many cases, dogs act as positive and active agents in their interactions with humans, and the process of positive and benign interactions can have a beneficial effect on both humans and dogs. However, not all interactions are friendly, and human society is often filled with stress, and this negativity can be contagious across species, such as dogs that chronically share their living environments with stressed-out owners. A number of studies have found that short-term acute stress is contagious in dog-human dyads, and in these studies, female dogs showed higher emotional sensitivity than male dogs and were strongly correlated with owner personality traits (Höglin et al., 2021; Sundman et al., 2019). In stress reduction activities for students on campus, specially trained dogs often act as therapy dogs and are used to alleviate a variety of stressors in students; however, these therapy dogs exhibit more stress-related behaviors during their interactions with students (Sarrafchi et al., 2022). Consequently, research on the transmission of stressful emotions in canines has called for an update of standardized policies and comprehensive guidelines for animal welfare.

When a human cried, even if it was pretending to cry, the dog responded by turning toward and approaching the human, whether the person crying was his owner or a stranger. Even when the crying stranger did not respond to the approaching dog, the dog did not turn his head to approach his owner for comfort. This finding was also confirmed in human infants, where dogs showed increased cortisol levels and both alert and submissive behaviors when the infants cried. Thus, the mechanism of emotional contagion triggers the dog's behavior when confronted with a crying human, rather than the dog's personal pain (egoistically driven by aversion and discomfort to the pain of others (Hortensius et al., 2016) driving the comforting behavior (Custance and Mayer, 2012).

Emotional contagion studies on animals have mostly been limited to conspecifics, and as the right-hand man of long-term human domestication, some researchers have focused their perspectives on emotional contagion in dog-human dyads and found interesting conclusions on the transmissibility of emotions across species. However, how do dogs recognize and respond to human emotions, a process in which visual, auditory and chemical signals act as a bridge to emotional contagion. Dogs can recognize human facial expressions and different emotional sounds for emotion recognition, which has been confirmed in many studies. A study found that even when half of the face is exposed, up or down, dogs can recognize emotions by associatively promoting to the other half of the face judging human expressions (Muller et al., 2015). Whereas when speakers were used to play non-emotional sounds (biological sounds such as crickets chirping and birds singing, or non-biological sounds such as rain and leaves rattling in the wind) and emotional sounds (negative crying or positive laughter), the dogs showed more behavioral responses to the latter and more freezing behaviors to the negative crying sounds (Huber et al., 2017). The dog's olfactory system has the amazing potential to collect sweat from the armpits of human subjects after watching videos that elicit fear or happiness, and when exposed to the odor of fear sweat, the dog recognizes the chemical signals of this fear and experiences a stress response of increased heart rate (D'Aniello et al., 2018).

### 2.4 Raven

Although the choice of the raven as a study subject is relatively unknown in the field of emotional contagion, the research conducted on it is an innovation in the field, not only because of the high sensitivity of these birds to the emotions of their own kind, but also because this type of research dissociates emotional contagion from behavioral contagion and proposes a new idea: behavioral contagion is not an antecedent to emotional contagion, a conclusion that overturns Hatfield's definition of emotional contagion as presupposed by mimicry of the behavior. However, it is important to be wary of differences in cognitive and behavioral styles due to species differences that can lead to differences in cognitive and behavioral styles, for example, the raven appeared to make pessimistic judgments in a cognitive bias task in a state of observing negative emotions in its own kind, but evidence of positive emotional contagion was only seen in young ravens in the context of social gaming (Wenig et al., 2021). Certain infectious behaviors, such as yawning and scratching, exist in humans and mammals, but there is no evidence that ravens exhibit any of the above behaviors (Gallup et al., 2022). Thus, it is likely that the process of emotional contagion in ravens lacks behavioral mimicry, but only emotional change. Therefore, the generalizability of the findings on emotional contagion among ravens to other species remains one of the many questions that need to be verified, and the role of behavioral mimicry in emotional contagion cannot be denied on the basis of a small number of studies, and the extant studies have some limitations (Vonk, 2019).

### 2.5 Nestor notabilis

Currently, only a handful of studies have experimented on the contagion of positive emotions, and the sensory cues for positive emotion contagion have mostly focused on hearing (Dietmar and Julia, 2005), as previous studies have found that deaf mice are less likely to play with their counterparts (Siviy and Panksepp, 1987). One of the strongest pieces of evidence for positive emotional contagion is a study of Nestor notabilis in which many birds began to play with non-playing conspecifics when Nestor notabilis play calls were played, rather than joining in a game that had already begun. This suggests that play calls can trigger positive emotional contagion in Nestor notabilis, and that such calls are not game invitations specific to a particular bird, but trigger collective play behavior (Schwing et al., 2017).

### 2.6 Elephant

Elephants are highly social animals that help the weaker members of their group and have close bonds with family members. Following distress events (intimidation or attack by conspecifics, social isolation, environmental threats, etc.), elephants become behaviorally agitated (e.g., ears forward, tail erect, movement) and emit agitated vocal signals (e.g., trumpets, roars and rumbles). When observing conspecifics that appear distressed, elephants will approach distressed individuals more often to emit a reassuringly distinctive sound (e.g., chirping) and to make physical contact to comfort them (e.g., touching genitals, mouth, head, etc.) (Nair et al., 2009). The phenomenon of elephant consolation has also been described in a number of anecdotal reports (Eranga et al., 2015; Iain et al., 2006; Sharma et al., 2020), but there have been no additional studies controlling for emotional contagion, and the scientific validity of these reports has been questioned.

### 2.7 Farm animal

Domestic pigs are a highly social class of farm animals, and in painful emotional states, pigs exhibit behaviors such as pain grunting, low tail posture (drooping or retracting tails), freezing, and avoidance. In positive moods, on the other hand, pigs indicate this byactively hanging tails, and playing (Iglesias and Camerlink, 2022; Reimert et al., 2013), and these behavioral indicators can help researchers to determine the emergence of their negative moods. Sensitivity to both positive and negative emotional contagion has been demonstrated in domestic pigs (Reimert et al., 2013), where salivary cortisol levels were measured and found that pigs were aroused by the events, whether they were rewarding events (pairs entering a reward gate containing straw, peat, and raisins for 5 min) or aversive events (entering aversive gates of 5 min social isolation accompanied by unpredictable stimuli). By observing the responses of demonstration pigs after experiencing rewarding and aversive events, naïve pigs showed more sniffing and more defecation behaviors in response to aversive gates, and showed more playful behaviors during rewarding events.

Piglets that had been previously restrained showed more intense fearful behaviors, such as decreased locomotion and increased freezing, when observing their peers being restrained, whereas merely witnessing the embarrassment of their peers being restrained in the past did not increase fearful responses when witnessing it again (Goumon and Spinka, 2016). The transmission of negative emotions can have an impact on the welfare of their communal rearing and, after being stimulated with different emotions, positively stimulated domestic pigs were more likely to elicit positive emotions from their naïve companions, whereas negative stimuli produced the opposite result (Inonge and Stephanie, 2017). In addition, domestic pigs are also characterized by contagious yawning (Norscia et al., 2021), and this ability to mimic automatically has been suggested as a possible prerequisite for emotional contagion.

Chemical signals released in the blood during goat slaughter can also lead to emotional contagion between conspecifics. Notably, normal blood-triggered olfactory or visual cues do not lead to emotional contagion of distress among goats, which only occurs when distressing emotions are generated during the slaughter process, leading to a stress response of elevated blood glucose (Ferguson and Warner, 2008; Kumar et al., 2023b,a). However, this conclusion is only based on a single study, which has not been repeated in other farm animals (such as pigs or chickens), and it has not been controlled whether stressors outside the slaughter environment (such as transportation or isolation) may trigger similar reactions. Such specific mechanisms need more cross-species verification.

Hens can become sensitized to the pain produced by their offspring, and when they perceive that their chicks are eating difficult food, they will alter their maternal feeding display (Christine and Stuart, 1996). When witnessing distressing emotions in chicks, hen vocalizations will increase, which is thought to be them calling the chicks away from dangerous situations, and the hen will less time preening and more walking (Edgar et al., 2013). When witnessing chicks accompanied by mild stress, domesticated hens also show behavioral consistency and socially-mediated arousal socially-mediated arousal (SMA) (Edgar and Nicol, 2018). SMA is a special form of emotional contagion, when witnessing behavioral or physiological changes in a kindred species that increase the observer's sensory alertness and attention to respond, such as decreased ground pecking and preening and increased freezing (Roelofs, 2017), and 19 subject- observer brood-pairings had reduced and positively correlated eye temperatures.

### 2.8 Zebrafish

The paucity of research on emotional contagion in fish may be related to their limited cognitive abilities and their limited response to injurious stimuli, resulting in fish having difficulty experiencing pain and not being able to recognize fear (James, 2002; Rose, 2014). The study of emotional contagion in fish has been a major challenge in the past few years. However, despite the difficulties, there are still some studies focusing on the phenomenon of emotional contagion in fish. Zebrafish, a hot vertebrate model organism in recent years, has high genomic homology with humans (Howe et al., 2013) and is widely used in toxicology studies (Zhang et al., 2020) and genetics studies (Knecht et al., 2017) due to its *in vitro* fertilization and embryonic transparency. When danger befalls, zebrafish increase their swimming speed, accompanied by freezing behavior and self-protection by diving down to the bottom (Kalueff et al., 2013), and these behaviors can indirectly help researchers to determine whether zebrafish are experiencing fearful emotions.

Citalopram is a selective serotonin reuptake inhibitor that reduces anxiety, and zebrafish that underwent citalopram-induced reductions in anxiety levels manifested themselves by moving away from the bottom of the tank and transmitting this low level of anxiety to other naïve individuals in the same tank (Burbano et al., 2021). Visual cues in zebrafish have been shown to act as conduits for emotional contagion. Upon spatial proximity to a predator, zebrafish elevate cortisol levels and remain underwater for extended periods of time to protect themselves, while other zebrafish, upon observing self-protective behavior in their own species, activate the same “defense mode” and activate the HPA axis to induce an increase in cortisol levels throughout the body, even when they are not exposed to the predator's environment (Oliveira et al., 2017). A few years later, this conclusion was confirmed in other researchers' manuscripts that zebrafish can be emotionally contagious through visual cues, and further studies found stronger levels of emotional contagion of fear within groups of zebrafish that were familiar with each other (after 7 days of co-hosting) compared to their unfamiliar counterparts (Priscila et al., 2019). New research finds that oxytocin plays a key role in fear conditioning in zebrafish (Akinrinade et al., 2023). Stimulated zebrafish release a certain chemical (Schreckstoff) into the water, and their counterparts, detecting this danger signal by smell, trigger protective behaviors of freezing and fast swimming, whereas individuals lacking oxytocin regulation are less sensitive to this chemical signal representing danger, and by injecting oxytocin succeed in increasing their freezing behaviors in the face of fear. An interesting point of view is that the underlying mechanisms of emotional contagion were retained in the genes of fish and mammals when they were still of the same ancestry, roughly before 450 million years ago (Deangelis and Hofmann, 2023).

Previously, emotional contagion was thought to be a unique ability of mammals and birds. However, the conclusion of the study of emotional contagion among zebrafish conspecifics proves that emotional contagion also exists in fish, which raises the question of whether highly socialized animals generally have the ability of emotional contagion, or whether emotional contagion is a special ability that determines the formation of socialization in conspecifics.

Emotional contagion was first studied in rodents (Anderson, 1939), and then in non-human primates (Nieuwburg et al., 2021; Laméris et al., 2020), dogs (Grigg et al., 2022), birds (Liévin-Bazin et al., 2018), elephants (Plotnik and de Waal, 2014), ravens (Wenig et al., 2021), and even zebrafish (Burbano et al., 2021), where similar emotional contagion has been found. This means that emotional contagion is not unique to humans, but is also present in lower animals. As one of the cornerstones of generative social behavior, the behavioral paradigm of emotional contagion has helped researchers understand the development of mental illness and further explore the functional mechanisms of cognitive behavior. However, the creation of emotional contagion can also lead to biased findings, particularly in animal experiments exploring emotion-related issues, pushing experimental results away from expectations. At present, the phenomenon of emotional contagion in animals is still an area to be explored and many key questions remain to be addressed.

## 3 Emotional contagion in humans

This section will discuss the manifestations and mechanisms of emotional contagion in human beings, especially the relationship between children's behavioral mimicry and emotional contagion. First of all, we will review the ability of infants and young children in emotional contagion, and then analyze the emotional contagion phenomenon of adults in physical and virtual space.

### 3.1 Emotional contagion in children

**Figure 1:**
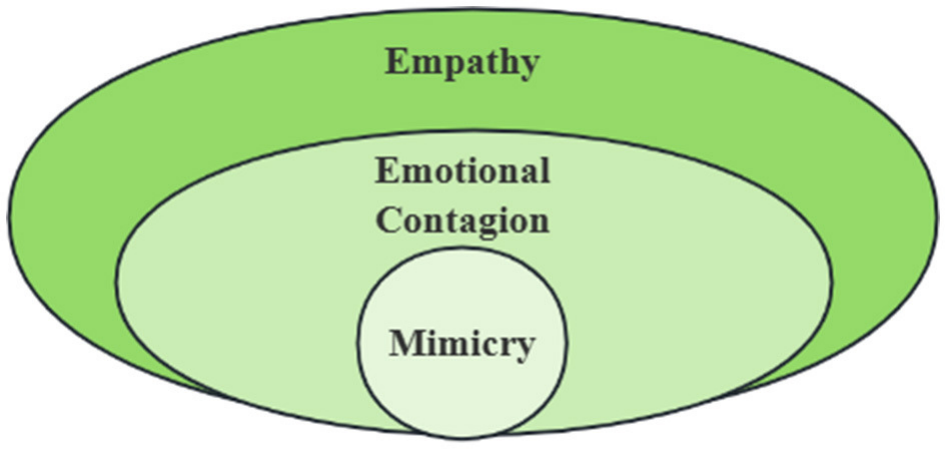
The Relationship between Mimicry, Emotional Contagion and Empathy. Mimicry, emotional contagion and empathy are progressive but independent concepts: **(1)**mimicry is a mechanical process in which individuals copy similar explicit behaviors (such as facial movements, postures or sounds), which may be independent of emotional experience; **(2)** Emotional contagion is a process in which individuals unconsciously synchronize other people's emotional states (such as facial expressions, sounds or physiological signals) to trigger their own emotional changes without cognitive understanding or intention; **(3)** Empathy contains the complex ability of cognitive and emotional components, which needs to distinguish others from their own emotional state, and may lead to targeted help behavior. Mimicry may be one of the mechanisms of emotional contagion, but emotional contagion does not necessarily lead to empathy (e.g., fear of contagion leads to flight rather than help); Empathy needs to be based on emotional contagion and superimposed with cognitive assessment (such as understanding others' situation).

Animals living in groups tend to learn information about their environment, whether positive or negative, from others through emotional contagion in order to ensure the continuation of their species. Humans are a highly social group, and the ability to recognize and respond to the emotions of others is a normal human ability, and this ability to transmit emotions binds individuals together, allowing them to become part of a social group and engage in pro-social behavior (Lakin and Chartrand, 2003; Lakin et al., 2008). However, we must clarify whether this capacity for emotional contagion is inherent in human beings, or whether it is something that they have acquired in their acquired existence, which will lead to the emergence of different paths in the future within the field of emotional contagion as well as higher levels of empathy, and affect the search for the mechanisms of certain psychiatric disorders (Figure 1). In order to address this issue, research related to emotional contagion in young human children is necessary.

Existing evidence continues to suggest that behavioral mimicry is one of the prerequisites for emotional contagion, and that mimicry of human facial behaviors is an important behavioral window for emotional contagion (Palagi et al., 2020). The use of soothers successfully disrupts this behavioral window, interfering with the caregiver's ability to mimic the infant's emotions, and diminishing the perception of either the infant's happy or painful emotions (Magdalena et al., 2014). Similarly, the disruption of the behavioral window by placing a pencil in the observer's mouth to prevent mimicry of low others' facial expressions impairs the observer's ability to recognize others' emotions (Birch-Hurst et al., 2022; Hirsch et al., 2023; Oberman et al., 2007). The induction of frowning muscle denervation by botulinum toxin resulted in reduced activation of the amygdala, an emotionally sensitive region, implying that alterations in facial expression modulate emotional centers through neural pathways (Hennenlotter et al., 2009), a study that sheds light on the physiological basis of behavioral mimicry in emotional contagion.

Yawning as a primitive stereotypical behavior has been shown to be contagious in non-human primates, and contagious yawning shares the same characteristics as emotionally contagious mimicry, i.e., both are rapid involuntary behavioral mimicry. Cordoni et al. observed 129 preschoolers (2.5 to 5.5 years) in five classrooms and found that the children who participated in the test were already capable of contagious yawning, were contagious to the yawns of others within 2 min of perceiving another person's spontaneous yawning, and that the level of spontaneous yawning was higher in boys than in girls, whereas the level of contagious yawning was not affected by gender Influence (Cordoni et al., 2021). Moving the life cycle forward, do neonates or toddlers also have the ability to be emotionally contagious? do neonates or toddlers also have the ability to transmit emotions? An interesting conclusion is that neonates start crying when they hear other neonates crying, but not when they hear their own cries, the cries of an older infant or the cries of a chimpanzee (Martin, 1982; Sagi, 1976; Simner, 1971). Does this mean that neonate is capable of emotional contagion? Ruffman et al. played four kinds of videos including infant crying, laughter, white noise and babbling to toddlers, young adults, and older adults respectively, and drew the following conclusions about toddlers' emotional contagion:(a) adults performed sadder than toddlers when watching crying videos; (b) regardless of age, they performed happier when watching laughter videos; and (c) toddlers performed comparably when watching crying videos and white noise videos. Ruffman suggests that toddlers' reactions to crying videos are more likely to be an aversive auditory stimulus than emotional contagion, as nearly half (42%) of toddlers did not show an emotional reaction to crying videos (compared to 45% of adults). However, this study does not deny the ability of toddlers to be emotionally contagious, but rather demonstrates a positive correlation between the level of emotional contagion and aging.

From a series of studies on human toddlers we can draw some conclusions that the occurrence of emotional contagion may need to be predicated on the mimicry of another person, through sensory channels such as visual (change in facial expression) or auditory (sound of crying) to induce imitative behaviors toward the other person, and consequently change the imitator's emotions. The process of emotional contagion in adult humans is even more complex because this behavioral paradigm occurs not only in real physical space, but also in virtual cyberspace, even if they are unable to observe others' physical behavior or facial expressions.

### 3.2 Emotional contagion in adults

#### 3.2.1 Physical space

The Framingham Heart Study was initiated in 1948 to prospectively investigate risk factors for cardiovascular disease, and involved four separate but related cohorts, all of which were socially related except for the Omni Cohort, which was enrolled in 1994. “The ‘Original Cohort'; the 'Offspring Cohort' (the children of the Original Cohort and the spouses of the children); and “Generation 3 Cohort” (the grandchildren of the Original Cohort). Despite the different aims of the study, these cohorts are a good vehicle for examining emotional contagion among members of the community. Fowler et al. chose 4,739 participants from the 1983 to 2003 Offspring Cohort and constructed a social network-based “mood map” using four items from the Center for Epidemiological Studies depression scale (CES-D) as indicators of happiness: I felt hopeful about the future; I was happy; enjoyed life; and I felt that I was just as good as other people. The four items of the Center for Epidemiological Studies Depression Scale (CES-D) (I felt hopeful about the future; I was happy; enjoyed life; I felt that I was just as good as other people) were used as indicators of wellbeing to construct a social network-based “mood map” (Fowler and Christakis, 2008). This study provides strong evidence for emotional contagion, where happy and unhappy people tend to appear in clusters in social networks, whether they are friends, spouses, siblings, co-workers or neighbors. And there is a limit to the extent of contagion of either positive or negative emotions, a limit that manifests itself in social relationships by extending up to three degrees of separation (for example, to one's friends' friends' friends). In terms of geographic proximity, friends living within a mile's distance and experiencing a happy mood were 25 per cent more likely to transmit a happy mood to others, compared with 14 per cent among siblings living within a mile's distance, compared with 8 per cent among cohabiting spouses, 34 per cent among next-door neighbors, and no such increase in the likelihood among co-workers. It is worth noting that negative emotions such as depression are more contagious in social networks, as negative emotions tend to mobilize others more. Rosenquist et al. Constructing datasets and network linkages after screening for depression by CES-D on a sample of 12,067 from the Original Cohort, Offspring Cohort, and other cohorts in the Framingham Heart Study Similar to the contagion of happiness, the contagion of depression appears to follow three degrees of separation, with friends and neighbors being closely related to the contagion of depression and depressed people losing 6% of their friends over a period of about 4 years. Healthy people who are surrounded by depressed people are twice as likely to be depressed compared to those who are surrounded by healthy people. And women who are depressed are more likely to spread this negativity to healthy people around them than men who are depressed (Rosenquist et al., 2011). Another study also suggests that there is a contagion of depression among adolescents as well (Schwartz-Mette and Rose, 2012). The spread of depression in social networks is closely related to emotional contagion, and current hypotheses suggest that depressed individuals induce inappropriate activation of the listener's MNS by way of emotional contagion when they confide negative emotions with others (Paz et al., 2022). In addition, differences in a combination of factors, such as the social environment in which an individual lives and personality traits, can affect his or her level of emotional susceptibility.

The workplace is often full of complex emotions. In the research field of organizational behavior, emotional factors are widely considered to have a significant and key impact on organizational effectiveness. Specifically, emotions play an important role in many core dimensions of work, including but not limited to job satisfaction, performance achievements and employees' psychological and physical well-being (Boukarras et al., 2024). Emotion, as a potential dynamic mechanism within an organization, affects employees' cognitive evaluation, motivation level and behavior choice, and then has a far-reaching chain reaction to the overall operation effect of the organization. Leaders are indispensable key figures in the organization, and their happiness or sadness will affect the emotional state of subordinates (Thomas and Nam, 2013). The infection of negative emotions in organizations will significantly reduce the efficiency of work and even produce workplace accidents (Laura and Valerio, 2021). In the medical workplace, the negative emotions conveyed by patients' language will reduce the professional commitment and workplace happiness of medical staff (Liu et al., 2021); in some challenging and stressful departments, such as intensive care unit, it is more important to protect nurses' emotional contagion sensitivity to improve their mental nursing ability (Koroglu and Oksuz, 2024). In an organization, emotional contagion is regulated by many factors, such as familiarity among employees (Magnier-Watanabe et al., 2024), personal internal intelligence level (Aksu et al., 2023) or leadership style (John et al., 2016). How to increase the infection of positive emotions in organizations and block or weaken the adverse effects of negative emotions is still an important direction to be studied.

#### 3.2.2 Virtual space

Emotional contagion through the online medium has become easier nowadays with the advancement of the Internet. Although it is difficult to observe the facial expressions and body movements of other users during communication through Online Social Networks (OSNs), emotional contagion can also be triggered among users by reading the contents of texts, photos or videos. For example, strong negative emotions when reading political messages (Sarah and Nicholas, 2019), or positive interactions with personal and emotional messages posted by politicians (Metz et al., 2019). In some Online Depression Communities (ODCs), the generation of negative emotions is extremely common, and this large-scale contagion of negative emotions is likely to cause community members to engage in risky behaviors. However, the original intention of these ODCs was to alleviate the psychological symptoms of participants in the community, but now they have the opposite result (Kwon and Cho, 2017). Tang et al. found in a Chinese ODC with 1,548 users that when negative emotions were present, such as posts containing negative emotions or even suicidal thoughts, expressions of fear and despair were more likely to appear in respondents' comments; this suggests that the presence of ODCs does not alleviate depression, but rather exacerbates depression levels. emotions and even suicidal thoughts, expressions of fear and despair were more likely to appear in the comments of respondents; this suggests that the presence of ODCs did not alleviate depression, but rather exacerbated depression levels. In addition, this study also found that positive emotions in ODC had higher levels of contagion than negative emotions (Tang et al., 2021). Compared with ODCs, participants in the diabetes community often post more positive content in their initial posts, but express less positive emotions in their replies, but there is no widespread emotional contagion in the diabetes community (Yao et al., 2022). In the online interactive community, patients with mental illness have more emotional contagion than patients with physical illness (Yao et al., 2022). In online communication, high arousal emotions such as awe, anger, and anxiety are transmitted at a higher level than low arousal emotions such as sadness (What, 2012), and depressed individuals tend to hide their thoughts in reality, while anonymous online communities act as emotional catharsis, and high arousal emotions such as fear tend to cause mass contagion.

Swearing is a high-arousal emotional category that has been little addressed in emotional contagion research. In face-to-face interpersonal interactions, the use of swear words increases informal perceptions of language and improves overall impressions of others (Nicoletta and Margherita, 2014). In anonymous public online communities, swearing is more related to emotional disinhibition and gaining the attention and approval of other users (Kwon and Cho, 2017). Kwon et al. demonstrate that overt first-level swearing (aka, parent) comments trigger second-level (aka, child) chains of interpersonal swearing by collecting offensive comments under the Trump ad series videos on YouTube (Anatoliy, 2017). Users who are attacked by offensive comments or who observe others interpersonal swearing may instead be swearing at specific or non-specific users, and such swearing can spread like an epidemic among online participants within a community context (Anatoliy, 2017).

Facebook, one of the largest OSNs in the world, houses more than 200 million users in different parts of the globe and has an extensive and persistent social network. Coviello et al. devised a method for detecting emotional contagion based on instrumental variable regression in economics and used rainfall as a source of variation affecting user sentiment in social networks (Liu et al., 2014). After analyzing the dataset for 1,180 days, it was found that rainy weather decreases positive sentiment posts by 1.19% and increases negative sentiment posts by 1.16%, and more importantly, for every increase in positive sentiment posts, the number of negative posts from friends (the user's Facebook friends) decreases by 1.80%, while for every increase in negative sentiment posts, the number of positive posts from friends decreased by 1.26 per cent (Coviello et al., 2014). This suggests that a user's personal emotional expression affects the emotional expression of his/her friends, which in turn generates both positive and negative user clusters in the social network. Further, in addition to the positive or negative emotions expressed by the content of the post, the number of likes, shares, followers and comments on the post were also used as an influencing factor, employing Amazon Mechanical Turk's list of word ratings that can be used to assess the happiness score of each word within the post to assess how much it influences the user's happiness, and accordingly to determine the most influential users in terms of happiness (Lin et al., 2023). These users are well known in the community and are constantly influencing the emotions of other users through their interactions. However, it should be noted that this kind of research mostly relies on text analysis (such as emotional vocabulary statistics), and may ignore the influence of non-verbal clues (such as emoticons or silent interaction). In addition, cultural differences between different platforms may be variables that need to be considered separately, and cross-platform reappearance research is urgently needed.

Emotional contagion in online communities should therefore be taken seriously by administrators, especially given the evidence that it is easier to get positive emotions to spread, and there should be human positive interventions in online communities such as ODCs and OSNs to prevent them from being the hardest hit by negative emotional contagion.

## 4 Theoretical models of emotional contagion

This section will introduce the theoretical model of emotional contagion, from sociological model to neurocognitive model. First, we will review the sociological model based on the infectious disease model, and then discuss the neurocognitive model and its role in explaining the emotional contagion mechanism (Table 1).

**Table 1:** Comparison between representative models.

**Types of models**	**Representative model**	**Advantage**	**Limitations**
Sociological models	SIS/SIR	Quantify the trend of group emotional contagion.	Suppose the emotional state is classified into two categories (such as positive/negative), ignoring the emotional complexity.
		It is suitable for predicting emotional contagion in large-scale social networks.	Individual neurological or psychological differences are not included.
Neurocognitive model	NMEC	Reveal the neural mechanism of behavioral mimicry and emotional contagion.	It is difficult to generalize to group dynamics.

### 4.1 Sociological models: insights from the infectious disease model SIS/SIR

Although it is said that the world does not have the same leaf, abstractly speaking, the process of spreading emotional contagion has a lot of similarities with the process of infecting infectious diseases, that is, both of them affect the individual's physiological or psychological conditions through other people (the transmission of infectious diseases must be contacted by both, while emotional contagion does not need) and gradually tend to synchronize the state of affairs. Half a century ago, Daley et al. applied mathematical models of infectious diseases to predict the spread of rumors (Daley and Kendall, 1964). And today, some extended models of epidemiological modeling are still used to explain the phenomenon of emotional transmission in populations.

The susceptible-infected-susceptible model (SIS model) is the most classical and simplest basic model of infectious diseases (Anderson and May, 1991), in which individuals have only two states: “susceptible” (not infected with the disease) and “infected” (infected with the disease). The susceptible-infected-recovered model (SIR model) is another classic model of infectious disease, in which the individual is in one of three states: “susceptible”, “infected” and “recovered” (the state of being immune to infection after contracting the disease). Currently, most sociological models describing the process of emotional contagion have evolved from the SIS/SIR model. Further, the propagation process of emotional contagion is more inclined to the SIRS model because the same emotion is repeated in the future (Dodds and Watts, 2005).

Hill et al. added a new term to the SIS model: a, representing uninfected individuals spontaneously infected at a constant rate a (Hill et al., 2010). In other words, in some cases susceptible individuals can also spontaneously become infected, meaning that they spontaneously develop certain emotions and infect other individuals. This contagion model, called SISa, can measure the scale of spread of emotional contagion because it can be applied to people who are not in contact in a social network. In common with other large-scale studies of emotional contagion, this study, which surveyed a participant population in the Framingham Heart Study and used the CES-D as an indicator of emotion, defined individuals who scored greater than 16 as the presence of discontent and those who received a score of 12 on the positively worded questions of the CES-D as content. Individuals presenting with discontent or content were defined as infected, and individuals with a neutral mood state were defined as susceptible (Hill indicated that individuals presenting with both content and discontent were less than 1/1,000, and therefore were not included in the mood states for this study). Based on this innovative SISa model, Hill successfully measured the average lifespan of contenet “infections” (10.1 years) and discontent “infections” (5 years) in the population and found that contenet had a greater rate of spontaneous infection than discontent, which had a greater rate of recovery (Hill et al., 2010). This means that the contagion level of positive emotions is higher than that of negative emotions and that individuals maintain the positive emotional state for a longer period of time, whereas negative emotions quickly return to the level of neutral emotions. Liu et al. further improved on the SISa model by discussing the contagion process of positive and negative emotions separately, i.e., susceptible-optimistic-susceptible (SOS) and susceptible-pessimistic-susceptible (SPS), and the SOSa-SPSa model similarly retained the automatic infection rate a (Liu et al., 2014). However, the stability of the equilibrium of the SOSa-SPSa model has not been mathematically tested, and its application is limited to the contagion of investor sentiment within the field of finance, which may be affected by factors such as the complex mechanisms of the financial markets, the complex characteristics of investors' social networks, etc. (Liu et al., 2014).

Although the epidemic-derived model described above can speculate the process of emotional contagion in a population, the biggest difference between emotional contagion and epidemic transmission is that emotional contagion, without the need for contact in reality, can be transmitted in virtual social networks. The stability of the SIS/SIR-derived model in the real space has been investigated, but the stability of its model in the virtual network needs to be further verified. Therefore, some researchers have linked cyberspace with physical space to explore the contagious effects of different emotions on individuals in dual space. Hong et al. coupled virtual and real space and constructed a personalized virtual and physical cyberspace-based emotional contagion model (PVP-ECM) based on the framework of the SIR model, which combines the emotional contagion in the physical and virtual space, arguing that an individual is subjected to simultaneous emotional contagion in both spaces (Hong et al., 2022). Another contribution of this study is the integration of different personalities with the process of emotional contagion, since different personality traits may lead to the production of opposite emotions when exposed to the same information. Although the effects of different personality traits on the intensity of emotional contagion have also been analyzed in previous studies (Stephen et al., 2011; Mengxiao et al., 2017; Lhommet et al., 2011), which examined the influence of different personality factors on the level of emotional contagion in virtual social networks (Hung et al., 2023), there are also researchers who have focused on the dual emotional contagion in both physical and cyberspace (Zhang et al., 2022). However, personalized models of emotional contagion grounded in both physical and virtual space after categorizing personality are still in the minority, e.g., emotional contagion of passengers under large-scale flight delays (Shao et al., 2021), control of emotional contagion of crowds during emergencies (Hong et al., 2020).

Cellular Automata (CA) is a discrete dynamical model within a finite grid, and due to its less computational effort but the ability to simulate complex spatio-temporal dynamic evolutions, improved models of CA have been applied to the evacuation of crowds in public places (Shaobo et al., 2009; Xingli et al., 2019). Emotional contagion is also characterized by spatio-temporal evolution, and combining CA with SIS/SIR model will further fit the real-world emotional contagion process. Fu et al. combined the CA approach based on the SIR model to simulate the dynamic emotional contagion process in a population and proposed the CA-SIRS emotional contagion model (Fu et al., 2014). Compared to the macroscopic SIR static model, Fu argues that the SIRS model better expresses the process of emotional contagion (Dodds and Watts, 2005), where recovering individuals are re-contagious to others' emotions after a certain period of time rather than reaching a state of emotional immunity, and combining this with CA produces an improved discrete model. Subsequently, simulations using the CA-SIRS model in a 100 × 100 cell space demonstrated that average crowd density and crowd motion were positively correlated with the level of emotional contagion, whereas reducing the average duration of emotional infections in the crowd significantly reduced the number of individuals who ended up being infected, with an adjustment in the average duration of the infected state from 300 to 50 resulting in a 25% reduction in the number of individuals who ended up being infected.

The SIS/SIR model and some of the extended models based on it reveal the process of emotional contagion among different groups of people, regardless of the existence of social networks, and provide several different types of theoretical frameworks, covering both physical and virtual spaces, with personality traits also being included in the modeling of emotional contagion. The contribution of these theoretical models not only sheds light on the process of emotional contagion among individuals, but also serves as a means of controlling emotions in populations during critical events, including blocking negative emotions and diffusing positive ones. However, although the above models have been proved to have good predictive efficacy, there are still some shortcomings. First, there are still some difficulties in applying and generalizing the models, as some of the models are limited to a specific scenario and lack external validation with larger samples. Second, there is still no uniform conclusion on which is the most scientific evaluation tool to measure emotional contagion, which also leads to the question of the validity of the models. Finally, unlike the objective transmission of viruses, the subjective experience of emotions can be facilitated or prevented by various unexpected factors in the real world.

### 4.2 Neurocognitive models: emotional contagion triggered by behavioral mimicry

Above, we have reviewed the evidence for infant behavioral mimicry, but we still need to learn more about the nature of mimicry. As part of the emotional contagion jigsaw, understanding how the mechanisms of mimicry work is crucial to a deeper exploration of emotional contagion.

Previous evidence has shown that infants can imitate the voices, physical behavior and facial expressions of others. Infants imitate by hearing their own and others' voices (John, 1993), and in comparisons of deaf infants with normal infants, it was found that deaf infants babble with delayed vocalizations and different durations of babble (Oller and Eilers, 1988; Oller et al., 1985). In twin boys, vocalization between boys with severe hearing loss and boys with normal hearing results in a progressively wider gap with age, with boys with hearing loss showing significant deficits in the use of vowels and consonants (Kent et al., 1987; Eilers and Oller, 1994). The mimicry of physical behaviors and sounds is perceptually transparent, or an intramodal comparisons (Meltzoff and Moore, 1997; Heyes and Ray, 2000), where infants can see or hear the behaviors or sounds of others, and at the same time they can see or hear their own physical movements or sounds thus continuously modifying the results of their mimicry. One of the dilemmas in developmental psychology is how infants imitate the facial expressions of others; if the mechanism of vocal mimicry is followed, infants are unable to see their own facial movements and cannot complete the mimicry process. Therefore, Meltzoff et al. proposed a new theoretical model to solve this problem, namely active intermodal mapping (AIM) (Meltzoff and Moore, 1997). Meltzoff sees mimicry as a process of matching to a target, in which infants are able to correct for differences between their own behavior and that of others, and argues that the perception and production of imitative behaviors are encoded within the same framework in the brain, and that this capacity for mimicry is encoded in the human brain without the need for an acquired learning process. However, a number of scholars have questioned the extent to which AIM is applicable, most notably the issues that AIM is justified when a sensory input is uniquely related to a cause, whereas the same behavior can express different meanings (Prochazkova and Kret, 2017; Kilner et al., 2007; Hickok, 2009), and there is no evidence to support the existence of AIM in tests with non-human primates (Hickok, 2009; Meltzoff and Decety, 2003).

Due to the various shortcomings exposed by AIM, Ray et al. proposed the associative sequence learning model (ASL), which improves on the shortcomings of AIM (Ray and Heyes, 2011). At the heart of ASL is the process of associative learning, the idea that stimuli in the environment will guide an infant's interactions with the world or with others, and that in the event of accidentally seeing another person make an action, the infant will make the same action at the same time. Seeing the action and making the same action is incidental, but there must be temporal coherence between the two and a matching vertical association will be established (Ray and Heyes, 2011). In contrast to AIM, Ray argues that mimicry is not innate, but is a sensory-motor learning process based on the socio-cultural environment (referred to as “the wealth of the stimulus”), which has been demonstrated in chimpanzee populations, where captive chimpanzees are more capable of mimicry than those fed by females alone.

However, ASL does not reveal the neural underpinnings of the perception-action process, nor the liaison channels through this. From the perspective of evolution, emotional contagion may be an instinctive reaction of human beings, which helps us to respond quickly and seek help when facing danger. The related research in neuroscience reveals the neural mechanism behind emotional contagion, such as the activation of mirror neuron system, etc. These perspectives provide a deeper explanation for us to propose a theoretical model of emotional contagion.

Therefore, Prochazkova et al. proposed a neurocognitive model of emotional contagion Neurocognitive Model of Emotional Contagion (NMEC) on the basis of the AIM and ASL models (Prochazkova and Kret, 2017). At the heart of NMEC is the Perception-Action Model Perception-Action Model (PAM), which assumes that perceiving the state of another person automatically activates representations in the observer that correspond to that state, thereby activating somatic and autonomic responses (Preston and de Waal, 2002), and that emotional contagion and behavioral mimicry depend on matching the PAM. In this, the MNS and ANS systems are thought to play a role, and PAM is more widespread than the mirror mechanism (Preston and de Waal, 2017). Further, the NMEC proposes possible neural substrates and neural circuits for automatic behavioral mimicry and emotional replication, which involves the synergistic action of behavioral mimicry and autonomic mimicry, and argues that autonomic mimicry, activation of the ANS, and emotional responses all evoke the same neural substrates and pathways in the brain. Connecting the MNS to the emotional system via the anterior insula completes the process of behavioral mimicry and emotional replication in emotional contagion. Remarkably, if NMEC's conclusions hold true, the ability for emotional contagion and even empathy is expected to emerge in future robots and enable true emotional communication.

## 5 Conclusion

Emotional contagion is an important building block for the emergence of empathy and one of the key conditions for the evolution of empathy in lower animals, and mimicry may be a prerequisite for inducing emotional contagion. From the conclusions of studies on zebrafish, emotional contagion may be a fairly ancient ability, engraved in the genes of different species. Since the process of emotional contagion cuts across many disciplines, including neuroscience, biology, psychology, and sociology, the study of emotional contagion is first and foremost a major challenge of interdisciplinary collaboration, and the research object is cross-species in nature, the place of occurrence covers both virtual and physical space, and the process of contagion presents a number of challenges, including the temporal and spatial nature. Yet none of these obstacles have stopped researchers from continuing to pursue the truth, and with the advent of imaging evidence, such as functional MRI, we can observe the subtle processes of emotional contagion with greater scientific rigor. And findings on emotional contagion in different species can be complemented to reinterpret the process of emotional contagion from different perspectives. The exploration of emotional contagion in virtual space, on the other hand, opens up new avenues of research and adds a variety of theoretical models of emotional contagion. One of the advantages of interdisciplinary research is that research tools from different fields can also complement each other, and theoretical models, whether they are developed from epidemiology, sociology, or neurobiology, have been developed to interpret the process of emotional contagion. Through systematic narration and multidisciplinary perspective, this paper discusses in detail the manifestations and mechanisms of emotional contagion in different animals and humans. It is found that emotional contagion is a common phenomenon, which has important evolutionary significance and social function. It is important to note that there is still a small body of research on positive emotional contagion in both humans and animals, which deserves to be further explored by more researchers in the future.

## References

[B1] Adriaense J. Martin J. S. Schiestl M. Lamm C. Bugnyar T. (2019). Reply to Vonk: disentangling emotional contagion from its underlying causes. Proc. Natl. Acad. Sci. U. S. A. 116, 18169–18170. 10.1073/pnas.1910556116 31431539 PMC6744921

[B2] Akinrinade I. Kareklas K. Teles M. C. Reis T. K. Gliksberg M. Petri G.. (2023). Evolutionarily conserved role of oxytocin in social fear contagion in zebrafish. Science 379, 1232–1237. 10.1126/science.abq5158 36952426

[B3] Aksu C. Ayar D. Kuslu S. (2023). The correlation of intrapersonal intelligence levels of nurses with their emotional contagion and caring behaviours. Appl. Nurs. Res. 73:151733. 10.1016/j.apnr.2023.151733 37722780

[B4] Anatoliy K. H. K. G. (2017). Is offensive commenting contagious online? Examining public vs interpersonal swearing in response to Donald Trump's YouTube campaign videos. Internet Res 27, 991–1010. 10.1108/IntR-02-2017-0072

[B5] Anderson E. E. (1939). The effect of the presence of a second animal upon emotional behavior in the male albino rat. J. Soc. Psychol. 10, 265–268. 10.1080/00224545.1939.9713365

[B6] Anderson M. May R. M. (1991). Infectious Diseases of Humans: Dynamics and Control. Oxford, UK: Oxford University Press.

[B7] Birch-Hurst K. Rychlowska M. Lewis M. B. Vanderwert R. E. (2022). Altering facial movements abolishes neural mirroring of facial expressions. Cogn. Affective Behav. Neurosci. 22, 316–327. 10.3758/s13415-021-00956-z 34642896 PMC8983526

[B8] Boukarras S. Ferri D. Borgogni L. Aglioti S. M. (2024). Neurophysiological markers of asymmetric emotional contagion: implications for organizational contexts. Front. Integr. Neurosci. 18:1321130. 10.3389/fnint.2024.1321130 38357225 PMC10861795

[B9] Boyko M. Kutz R. Grinshpun J. Zvenigorodsky V. Gruenbaum S. E. Gruenbaum B. F.. (2015). Establishment of an animal model of depression contagion. Behav. Brain. Res. 281, 358–363. 10.1016/j.bbr.2014.12.017 25523029 PMC4305483

[B10] Bresciani C. Cordoni G. Palagi E. (2022). Playing together, laughing together: rapid facial mimicry and social sensitivity in lowland gorillas. Curr. Zool. 68, 560–569. 10.1093/cz/zoab092 36324534 PMC9616060

[B11] Burbano L. D. Macri S. Porfiri M. (2021). Collective emotional contagion in zebrafish. Front. Behav. Neurosci. 15:730372. 10.3389/fnbeh.2021.730372 34566596 PMC8458645

[B12] Carnevali L. Montano N. Tobaldini E. Thayer J. F. Sgoifo A. (2020). The contagion of social defeat stress: insights from rodent studies. Neurosci. Biobehav. Rev. 111, 12–18. 10.1016/j.neubiorev.2020.01.011 31931035

[B13] Christine J. N. Stuart J. P. (1996). The maternal feeding display of domestic hens is sensitive to perceived chick error. Anim. Behav. 52, 767–774. 10.1006/anbe.1996.0221

[B14] Church R. M. (1959). Emotional reactions of rats to the pain of others. J. Comp. Physiol. Psychol. 52, 132–134. 10.1037/h0043531 13654562

[B15] Cordoni G. Favilli E. Palagi E. (2021). Earlier than previously thought: yawn contagion in preschool children. Dev. Psychobiol. 63, 931–944. 10.1002/dev.22094 33506489

[B16] Coviello L. Sohn Y. Kramer A. D. I. Marlow C. Franceschetti M. Christakis N. A.. (2014). Detecting emotional contagion in massive social networks. PLoS One 9:e90315. 10.1371/journal.pone.0090315 24621792 PMC3951248

[B17] Custance D. Mayer J. (2012). Empathic-like responding by domestic dogs (Canis familiaris) to distress in humans: an exploratory study. Anim. Cogn. 15, 851–9. 10.1007/s10071-012-0510-1 22644113

[B18] Daley D. J. Kendall D. G. (1964). Epidemics and rumours. Nature 204:1118. 10.1038/2041118a0 14243408

[B19] D'Aniello B. Semin G. R. Alterisio A. Aria M. Scandurra A. (2018). Interspecies transmission of emotional information via chemosignals: from humans to dogs (*Canis lupus* familiaris). Anim. Cogn. 21, 67–78. 10.1007/s10071-017-1139-x 28988316

[B20] Davila R. M. Menzler S. Zimmermann E. (2008). Rapid facial mimicry in orangutan play. Biol. Lett. 4, 27–30. 10.1098/rsbl.2007.0535 18077238 PMC2412946

[B21] Deangelis R. S. Hofmann H. A. (2023). The spread of fear in an empathetic fish. Science 379, 1186–1187. 10.1126/science.adh0769 36952409

[B22] Dezecache G. Jacob P. Grèzes J. (2015). Emotional contagion: its scope and limits. Trends. Cogn. Sci. 19, 297–299. 10.1016/j.tics.2015.03.011 25891260

[B23] Dietmar T. Julia V. (2005). Human laughter, social play, and play vocalizations of non-human primates: an evolutionary approach. Behaviour 142, 217–240. 10.1163/156853905362764021355640

[B24] Dodds P. S. Watts D. J. A. (2005). generalized model of social and biological contagion. J. Theor. Biol. 232, 587–604. 10.1016/j.jtbi.2004.09.006 15588638

[B25] Edgar J. L. Nicol C. J. (2018). Socially-mediated arousal and contagion within domestic chick broods. Sci. Rep. 8:10509. 10.1038/s41598-018-28923-8 30002482 PMC6043517

[B26] Edgar J. L. Paul E. S. Nicol C. J. (2013). Protective mother hens: cognitive influences on the avian maternal response. Anim. Behav. 86, 223–229. 10.1016/j.anbehav.2013.05.004

[B27] Eilers R. E. Oller D. K. (1994). Infant vocalizations and the early diagnosis of severe hearing impairment. J. Pediatr. 124, 199–203. 10.1016/S0022-3476(94)70303-5 8301422

[B28] Eranga R. Ashoka D. G. R. Koun S. (2015). Tourism-induced disturbance of wildlife in protected areas: a case study of free ranging elephants in Sri Lanka. Glob. Ecol. Conserv. 4, 625–631. 10.1016/j.gecco.2015.10.013

[B29] Feneran A. N. O'Donnell R. Press A. Yosipovitch G. Cline M. Dugan G. . (2013). Monkey see, monkey do: contagious itch in nonhuman primates. Acta. Derm. Venereol. 93, 27–29. 10.2340/00015555-1406 22735614

[B30] Ferguson D. M. Warner R. D. (2008). Have we underestimated the impact of pre-slaughter stress on meat quality in ruminants? Meat. Sci. 80, 12–19. 10.1016/j.meatsci.2008.05.004 22063165

[B31] Ferretti V. Papaleo F. (2019). Understanding others: emotion recognition in humans and other animals. Genes Brain Behav. 18:e12544. 10.1111/gbb.12544 30549185

[B32] Fowler J. H. Christakis N. A. (2008). Dynamic spread of happiness in a large social network: longitudinal analysis over 20 years in the Framingham heart study. BMJ 337:a2338. 10.1136/bmj.a2338 19056788 PMC2600606

[B33] Fu L. Song W. Lv W. Lo S. (2014). Simulation of emotional contagion using modified SIR model: a cellular automaton approach. Physica A 405, 380–391. 10.1016/j.physa.2014.03.043

[B34] Gallup A. C. (2021). On the link between emotional contagion and contagious yawning. Neurosci. Biobehav. Rev. 121, 18–19. 10.1016/j.neubiorev.2020.11.023 33271163

[B35] Gallup A. C. Schild A. B. Uhlein M. A. Bugnyar T. Massen J. (2022). No evidence for contagious yawning in juvenile ravens (*Corvus corax*): an observational study. Animals 12:1357. 10.3390/ani12111357 35681822 PMC9179381

[B36] Goumon S. Spinka M. (2016). Emotional contagion of distress in young pigs is potentiated by previous exposure to the same stressor. Anim. Cogn. 19, 501–511. 10.1007/s10071-015-0950-5 26753689

[B37] Grigg E. K. Liu S. Dempsey D. G. Wong K. Bain M. Sollers J. J.. (2022). Assessing the relationship between emotional states of dogs and their human handlers, using simultaneous behavioral and cardiac measures. Front. Vet. Sci. 9:897287. 10.3389/fvets.2022.897287 35898554 PMC9310693

[B38] Hale J. Hamilton A. F. (2016). Testing the relationship between mimicry, trust and rapport in virtual reality conversations. Sci. Rep. 6:35295. 10.1038/srep35295 27739460 PMC5064448

[B39] Harr A. L. Gilbert V. R. Phillips K. A. (2009). Do dogs (*Canis familiaris*) show contagious yawning? Anim. Cogn. 12, 833–837. 10.1007/s10071-009-0233-0 19452178

[B40] Hatfield E. Cacioppo J. T. Rapson R. L. (1994). Emotional Contagion. Cambridge: Cambridge University Press. 10.1017/CBO9781139174138

[B41] Hennenlotter A. Dresel C. Castrop F. Ceballos-Baumann A. O. Wohlschlager A. M. Haslinger B.. (2009). The link between facial feedback and neural activity within central circuitries of emotion—new insights from botulinum toxin-induced denervation of frown muscles. Cereb. Cortex. 19, 537–542. 10.1093/cercor/bhn104 18562330

[B42] Hernandez-Lallement J. Gómez-Sotres P. Carrillo M. (2022). Towards a unified theory of emotional contagion in rodents-a meta-analysis. Neurosci. Biobehav. Revi. 132, 1229–1248. 10.1016/j.neubiorev.2020.09.010 33022297

[B43] Heyes C. M. Ray E. D. (2000). What is the significance of imitation in animals? Adv. Study Behav. 29, 215–245. 10.1016/S0065-3454(08)60106-05377343

[B44] Hickok G. (2009). Eight problems for the mirror neuron theory of action understanding in monkeys and humans. J. Cogn. Neurosci. 21, 1229–1243. 10.1162/jocn.2009.21189 19199415 PMC2773693

[B45] Hill A. L. Rand D. G. Nowak M. A. Christakis N. A. (2010). Emotions as infectious diseases in a large social network: the SISa model. Proc. Biol. Sci. 277, 3827–3835. 10.1098/rspb.2010.1217 20610424 PMC2992714

[B46] Hirsch J. Zhang X. Noah J. A. Bhattacharya A. (2023). Neural mechanisms for emotional contagion and spontaneous mimicry of live facial expressions. Philos. Trans. R. Soc. B. 378:20210472. 10.1098/rstb.2021.0472 36871593 PMC9985973

[B47] Höglin A. Van Poucke E. Katajamaa R. Jensen P. Theodorsson E. Roth L. S. V.. (2021). Long-term stress in dogs is related to the human-dog relationship and personality traits. Sci. Rep. 11:8612. 10.1038/s41598-021-88201-y 33883667 PMC8060293

[B48] Hong X. Zhang G. Lu D. (2020). Control strategies for crowd emotional contagion coupling the virtual and physical cyberspace in emergencies. IEEE Access 8, 37712–37726. 10.1109/ACCESS.2020.2975808

[B49] Hong X. Zhang G. Lu D. Liu H. Zhu L. Xu M.. (2022). Personalized crowd emotional contagion coupling the virtual and physical cyberspace. IEEE Trans. Syst. Man. Cybern. Syst. 52, 1638–1652. 10.1109/TSMC.2020.3034395

[B50] Hortensius R. Schutter D. J. de Gelder B. (2016). Personal distress and the influence of bystanders on responding to an emergency. Cogn. Affect. Behav. Neurosci. 16, 672–688. 10.3758/s13415-016-0423-6 27126708 PMC4949296

[B51] Howe K. Clark M. D. Torroja C. F. Torrance J. Berthelot C. Muffato M.. (2013). The zebrafish reference genome sequence and its relationship to the human genome. Nature 496, 498–503. 10.1038/nature12111 23594743 PMC3703927

[B52] Huber A. Barber A. Farago T. Muller C. A. Huber L. (2017). Investigating emotional contagion in dogs (Canis familiaris) to emotional sounds of humans and conspecifics. Anim. Cogn. 20, 703–715. 10.1007/s10071-017-1092-8 28432495 PMC5486498

[B53] Hung C. Gao X. Liu Z. Chai Y. Liu T. Liu C. C. E. C. M.. (2023). A cognitive emotional contagion model in social networks. Multimed Tools Appl. 83, 1001–1023. 10.1007/s11042-023-15394-x

[B54] Iain D. Shivani B. George W. Fritz V. (2006). Behavioural reactions of elephants towards a dying and deceased matriarch. Appl. Anim. Behav. Sci. 100, 87–102. 10.1016/j.applanim.2006.04.014

[B55] Iglesias P. M. Camerlink I. (2022). Tail posture and motion in relation to natural behaviour in juvenile and adult pigs. Animal 16:100489. 10.1016/j.animal.2022.100489 35334394

[B56] Inonge R. Stephanie F. T. BR, J. EB. (2017). Emotional states and emotional contagion in pigs after exposure to a positive and negative treatment. Appl. Anim. Behav. Sci. 193, 37–42. 10.1016/j.applanim.2017.03.009

[B57] James D. R. (2002). The neurobehavioral nature of fishes and the question of awareness and pain. Rev. Fish. Sci. 10, 1–38. 10.1080/20026491051668

[B58] John A. Nicolas B. Philippe J. Boas S. (2016). Charisma: an ill-defined and ill-measured gift. Annu. Rev. Organ. Psychol. Organ. Behav. 3, 293–319. 10.1146/annurev-orgpsych-041015-062305

[B59] John L. L. (1993). The Child's Path to Spoken Language. Cambridge, MA: Harvard University Press.

[B60] Joly-Mascheroni R. M. Senju A. Shepherd A. J. (2008). Dogs catch human yawns. Biol. Lett. 4, 446–448. 10.1098/rsbl.2008.0333 18682357 PMC2610100

[B61] Kalueff A. V. Gebhardt M. Stewart A. M. Cachat J. M. Brimmer M. Chawla J. S.. (2013). Towards a comprehensive catalog of zebrafish behavior 1.0 and beyond. Zebrafish 10, 70–86. 10.1089/zeb.2012.0861 23590400 PMC3629777

[B62] Kavaliers M. Choleris E. Colwell D. D. (2001). Learning from others to cope with biting flies: social learning of fear-induced conditioned analgesia and active avoidance. Behav. Neurosci. 115, 661–674. 10.1037/0735-7044.115.3.661 11439455

[B63] Kent R. D. Osberger M. J. Netsell R. Hustedde C. G. (1987). Phonetic development in identical twins differing in auditory function. J. Speech Hear. Disord. 52, 64–75. 10.1044/jshd.5201.64 3807347

[B64] Kilner J. M. Friston K. J. Frith C. D. (2007). Predictive coding: an account of the mirror neuron system. Cogn. Process 8, 159–166. 10.1007/s10339-007-0170-2 17429704 PMC2649419

[B65] Knecht A. L. Truong L. Marvel S. W. Reif D. M. Garcia A. Lu C.. (2017). Transgenerational inheritance of neurobehavioral and physiological deficits from developmental exposure to benzo[a]pyrene in zebrafish. Toxicol. Appl. Pharmacol. 329, 148–157. 10.1016/j.taap.2017.05.033 28583304 PMC5539966

[B66] Koroglu S. Oksuz E. (2024). How emotional contagion shapes spiritual care competence: insights from a cross-sectional study on intensive care nurses. Nurs. Crit. Care 29, 1394–1404. 10.1111/nicc.13160 39318081

[B67] Kumar P. Abubakar A. A. Ahmed M. A. Hayat M. N. Ajat M. Kaka U.. (2023a). Electroencephalogram and physiological responses as affected by slaughter empathy in goats. Animals 13:1100. 10.3390/ani13061100 36978640 PMC10044356

[B68] Kumar P. Ahmed M. A. Abubakar A. A. Hayat M. N. Kaka U. Ajat M.. (2023b). Improving animal welfare status and meat quality through assessment of stress biomarkers: a critical review. Meat. Sci. 197:109048. 10.1016/j.meatsci.2022.109048 36469986

[B69] Kwon K. H. Cho D. (2017). Swearing effects on citizen-to-citizen commenting online : a large-scale exploration of political versus nonpolitical online news sites. Soc. Sci. Comput. Rev. 35, 84–102. 10.1177/0894439315602664

[B70] Lakin J. L. Chartrand T. L. (2003). Using nonconscious behavioral mimicry to create affiliation and rapport. Psychol. Sci. 14, 334–339. 10.1111/1467-9280.14481 12807406

[B71] Lakin J. L. Chartrand T. L. Arkin R. M. I. (2008). am too just like you: nonconscious mimicry as an automatic behavioral response to social exclusion. Psychol. Sci. 19, 816–822. 10.1111/j.1467-9280.2008.02162.x 18816290

[B72] Laméris D. W. Berlo E. Sterck E. H. M. Bionda T. Kret M. E. (2020). Low relationship quality predicts scratch contagion during tense situations in orangutans (*Pongo pygmaeus*). *Am. J. Primatol*. 82:e23138. 10.1002/ajp.23138 32333423 PMC7379188

[B73] Langford D. J. Crager S. E. Shehzad Z. Smith S. B. Sotocinal S. G. Levenstadt J. S.. (2006). Social modulation of pain as evidence for empathy in mice. Science 312, 1967–1970. 10.1126/science.1128322 16809545

[B74] Laura P. Valerio M. P. T. G Claudio B. (2021). Emotional contagion as a trigger for moral disengagement: their effects on workplace injuries. Saf. Sci. 140:105317. 10.1016/j.ssci.2021.105317

[B75] Lhommet M. Lourdeaux D. Barthès J.-P. (2011). “Never alone in the crowd: a microscopic crowd model based on emotional contagion,” in 2011 IEEE/WIC/ACM International Conferences on Web Intelligence and Intelligent Agent Technology, Lyon, France (IEEE), pp. 89–92. 10.1109/WI-IAT.2011.149

[B76] Liévin-Bazin A. Pineaux M. Clerc O. Gahr M. von Bayern A. M. P. Bovet D.. (2018). Emotional responses to conspecific distress calls are modulated by affiliation in cockatiels (*Nymphicus hollandicus*). *PLoS ONE* 13:e0205314. 10.1371/journal.pone.0205314 30300404 PMC6177178

[B77] Lin C. Predicting L. i. Y. (2023). happiness contagion on online social networks. Multimed. Tools Appl. 82, 2821–2838. 10.1007/s11042-022-11989-y24621792

[B78] Liu B. Zhu N. Wang H. Li F. Men C. (2021). Protecting nurses from mistreatment by patients: a cross-sectional study on the roles of emotional contagion susceptibility and emotional regulation ability. Int. J. Environ. Res. Public Health 18:6331. 10.3390/ijerph18126331 34208160 PMC8296175

[B79] Liu Z. Zhang T. Lan Q. (2014). An extended SISa model for sentiment contagion. Discrete Dyn. Nat. Soc. 2014, 1–7. 10.1155/2014/262384

[B80] Lu J. S. Chen Q. Y. Zhou S. B. Wu F. Y. Liu R. H. Zhou Z. X.. (2019). Contagious itch can be induced in humans but not in rodents. Mol. Brain 12:38. 10.1186/s13041-019-0455-2 31014383 PMC6480616

[B81] Madsen E. A. Persson T. (2013). Contagious yawning in domestic dog puppies (*Canis lupus* familiaris): the effect of ontogeny and emotional closeness on low-level imitation in dogs. Anim. Cogn. 16, 233–240. 10.1007/s10071-012-0568-9 23076724

[B82] Magdalena R. Sebastian K. Markus B. Sylvie D. Maria A. Leah Z.. (2014). Pacifiers disrupt adults' responses to infants' emotions. Basic Appl. Soc. Psych. 36, 299–308. 10.1080/01973533.2014.915217

[B83] Magnier-Watanabe R. Benton C. Orsini P. Uchida T. (2024). Familiarity as main predictors of emotional contagion at work in Japan. Int. J. Organ. Anal. 33, 780–806. 10.2139/ssrn.5177038

[B84] Martin Grace B, and Clark, R. D.. (1982). Distress crying in neonates: species and peer specificity. Dev. Psychol. 18, 3–9. 10.1037/0012-1649.18.1.3

[B85] Meade G. M. Charron L. S. Kilburn L. W. Pei Z. Wang H. Robinson S. A.. (2021). model of negative emotional contagion between male-female rat dyads: effects of voluntary exercise on stress-induced behavior and BDNF-TrkB signaling. Physiol. Behav. 234:113286. 10.1016/j.physbeh.2020.113286 33321142 PMC8536152

[B86] Meeuwis S. H. Skvortsova A. van Laarhoven A. Holle H. Evers A. (2022). Can contagious itch be affected by positive and negative suggestions? Exp. Dermatol. 31, 1853–1862. 10.1111/exd.14663 36048562 PMC10087404

[B87] Meltzoff A. N. Decety J. (2003). What imitation tells us about social cognition: a rapprochement between developmental psychology and cognitive neuroscience. Philos. Trans. R. Soc. Lond. B. Biol. Sci. 358, 491–500. 10.1098/rstb.2002.1261 12689375 PMC1351349

[B88] Meltzoff A. N. Moore M. K. (1997). Explaining facial imitation: a theoretical model. Early Dev. Parent. 6, 179–192. 10.1002/(SICI)1099-0917(199709/12)6:3/4<179::AID-EDP157>3.0.CO;2-R 24634574 PMC3953219

[B89] Mengxiao C. Guijuan Z. Mengsi W. Dianjie L. Hong L. (2017). A method of emotion contagion for crowd evacuation. Phys. A Stat. Mech. Appl. 483, 250–258. 10.1016/j.physa.2017.04.13733600340

[B90] Metz M. Kruikemeier S. Lecheler S. (2019). Personalization of politics on Facebook: examining the content and effects of professional, emotional and private self-personalization. Inf. Commun. Soc. 23, 1481–1498. 10.1080/1369118X.2019.1581244

[B91] Muller C. A. Schmitt K. Barber A. L. Huber L. (2015). Dogs can discriminate emotional expressions of human faces. Curr. Biol. 25, 601–605. 10.1016/j.cub.2014.12.055 25683806

[B92] Nair S. Balakrishnan R. Seelamantula C. S. Sukumar R. (2009). Vocalizations of wild Asian elephants (*Elephas maximus*): structural classification and social context. J. Acoust. Soc. Am. 126, 2768–2778. 10.1121/1.3224717 19894852

[B93] Nakahashi W. Ohtsuki H. (2015). When is emotional contagion adaptive? J. Theor. Biol. 380, 480–488. 10.1016/j.jtbi.2015.06.014 26113192

[B94] Nakahashi W. Ohtsuki H. (2018). Evolution of emotional contagion in group-living animals. J. Theor. Biol. 440, 12–20. 10.1016/j.jtbi.2017.12.015 29253506

[B95] Nakayama K. (2004). Observing conspecifics scratching induces a contagion of scratching in Japanese monkeys (*Macaca fuscata*). J. Comp. Psychol. 118, 20–24. 10.1037/0735-7036.118.1.20 15008669

[B96] Nicoletta C. Margherita G. (2014). Swearing in political discourse. J. Lang. Soc. Psychol. 33, 537–547. 10.1177/0261927X14533198

[B97] Nieuwburg E. G. I. Ploeger A. Kret M. E. (2021). Emotion recognition in nonhuman primates: how experimental research can contribute to a better understanding of underlying mechanisms. Neurosci. Biobeha. Rev. 123, 24–47. 10.1016/j.neubiorev.2020.11.029 33453306

[B98] Norscia I. Coco E. Robino C. Chierto E. Cordoni G. (2021). Yawn contagion in domestic pigs (*Sus scrofa*). *Sci. Rep*. 11:1851. 10.1038/s41598-020-80545-1 33473157 PMC7817675

[B99] Oberman L. M. Winkielman P. Ramachandran V. S. (2007). Face to face: blocking facial mimicry can selectively impair recognition of emotional expressions. Soc. Neurosci. 2, 167–178. 10.1080/17470910701391943 18633815

[B100] O'Hara S. J. Reeve A. V. (2011). A test of the yawning contagion and emotional connectedness hypothesis in dogs, Canis familiaris. Anim. Behav. 81, 335–340. 10.1016/j.anbehav.2010.11.005

[B101] Oliveira T. A. Idalencio R. Kalichak F. Dos S. R. J. Koakoski G. Abreu d. e.. (2017). MS, et al. Stress responses to conspecific visual cues of predation risk in zebrafish. PeerJ 5:e3739. 10.7717/peerj.3739 28890851 PMC5588784

[B102] Oller D. K. Eilers R. E. (1988). The role of audition in infant babbling. Child Dev. 59, 441–449. 10.2307/1130323 3359864

[B103] Oller D. K. Eilers R. E. Bull D. H. Carney A. E. (1985). Prespeech vocalizations of a deaf infant: a comparison with normal metaphonological development. J. Speech Hear. Res. 28, 47–63. 10.1044/jshr.2801.47 3981997

[B104] Palagi E. Celeghin A. Tamietto M. Winkielman P. Norscia I. (2020). The neuroethology of spontaneous mimicry and emotional contagion in human and non-human animals. Neurosci. Biobehav. Rev. 111, 149–165. 10.1016/j.neubiorev.2020.01.020 31972204

[B105] Palagi E. Nicotra V. Cordoni G. (2015). Rapid mimicry and emotional contagion in domestic dogs. R. Soc. Open Sci. 2:150505. 10.1098/rsos.150505 27019737 PMC4807458

[B106] Palagi E. Norscia I. (2011). Scratching around stress: hierarchy and reconciliation make the difference in wild brown lemurs (*Eulemur fulvus*). Stress 14, 93–97. 10.3109/10253890.2010.505272 20666657

[B107] Parr L. A. Waller B. M. Vick S. J. Bard K. A. (2007). Classifying chimpanzee facial expressions using muscle action. Emotion 7, 172–181. 10.1037/1528-3542.7.1.172 17352572 PMC2826116

[B108] Paz L. V. Viola T. W. Milanesi B. B. Sulzbach J. H. Mestriner R. G. Wieck A.. (2022). Contagious depression: automatic mimicry and the mirror neuron system—a review. Neurosci. Biobehav. Rev. 134:104509. 10.1016/j.neubiorev.2021.12.032 34968526

[B109] Perez-Manrique A. Gomila A. (2022). Emotional contagion in nonhuman animals: a review. Wiley. Interdiscip. Rev. Cogn. Sci. 13:e1560. 10.1002/wcs.1560 33951303 PMC9285817

[B110] Pisansky M. T. Hanson L. R. Gottesman I. I. Gewirtz J. C. (2017). Oxytocin enhances observational fear in mice. Nat. Commun. 8:2102. 10.1038/s41467-017-02279-5 29235461 PMC5727393

[B111] Plotnik J. M. de Waal F. B. M. (2014). Asian elephants (*Elephas maximus*) reassure others in distress. PeerJ 2:e278. 10.7717/peerj.278 24688856 PMC3932735

[B112] Preston S. D. de Waal F. (2017). Only the PAM explains the personalized nature of empathy. Nat. Rev. Neurosci. 18:769. 10.1038/nrn.2017.140 29097789

[B113] Preston S. D. de Waal F. B. (2002). Empathy: its ultimate and proximate bases. Behav. Brain Sci. 25, 20–71. 10.1017/S0140525X02000018 12625087

[B114] Priscila F. S. Carlos G. D. L. Ana C. L. (2019). Fear contagion in zebrafish: a behaviour affected by familiarity. Anim. Behav. 153, 95–103. 10.1101/521187

[B115] Prochazkova E. Kret M. E. (2017). Connecting minds and sharing emotions through mimicry: a neurocognitive model of emotional contagion. Neurosci. Biobehav. Rev. 80, 99–114. 10.1016/j.neubiorev.2017.05.013 28506927

[B116] Qu Y. Zhang L. An S. Tai F. Qiao H. (2023). Chronic stress and stressful emotional contagion affect the empathy-like behavior of rats. Cogn. Affect. Behav. Neurosci. 23, 1160–1174. 10.3758/s13415-023-01081-9 36899132

[B117] Ray E. Heyes C. (2011). Imitation in infancy: the wealth of the stimulus. Dev. Sci. 14, 92–105. 10.1111/j.1467-7687.2010.00961.x 21159091

[B118] Reimert I. Bolhuis J. E. Kemp B. Rodenburg T. B. (2013). Indicators of positive and negative emotions and emotional contagion in pigs. Physiol. Behav. 109, 42–50. 10.1016/j.physbeh.2012.11.002 23159725

[B119] Roelofs K. (2017). Freeze for action: neurobiological mechanisms in animal and human freezing. Philos. Trans. R. Soc. Lond. B. Biol. Sci. 372:20160206. 10.1098/rstb.2016.0206 28242739 PMC5332864

[B120] Rose J. D. A. R. (2014). Can fish really feel pain? Fish Fish 15, 97–133. 10.1111/faf.12010

[B121] Rosenquist J. N. Fowler J. H. Christakis N. A. (2011). Social network determinants of depression. Mol. Psychiatry 16, 273–281. 10.1038/mp.2010.13 20231839 PMC3832791

[B122] Sagi Abraham, and Hoffman, M. L.. (1976). Empathic distress in the newborn. Dev. Psychol. 12, 175–176. 10.1037/0012-1649.12.2.175

[B123] Saito Y. Yuki S. Seki Y. Kagawa H. Okanoya K. (2016). Cognitive bias in rats evoked by ultrasonic vocalizations suggests emotional contagion. Behav. Processes 132, 5–11. 10.1016/j.beproc.2016.08.005 27591850

[B124] Sarah S. Nicholas B. (2019). Why keep arguing? Predicting engagement in political conversations online. Sage Open 9, 1–13. 10.1177/215824401982885034290901

[B125] Sarrafchi A. David-Steel M. Pearce S. D. de Zwaan N. Merkies K. (2022). Effect of human-dog interaction on therapy dog stress during an on-campus student stress buster event. Appl. Anim. Behav. Sci. 253:105659. 10.1016/j.applanim.2022.105659

[B126] Schwartz-Mette R. A. Rose A. J. (2012). Co-rumination mediates contagion of internalizing symptoms within youths' friendships. Dev. Psychol. 48, 1355–1365. 10.1037/a0027484 22369336 PMC3371303

[B127] Schwing R. Nelson X. J. Wein A. Parsons S. (2017). Positive emotional contagion in a New Zealand parrot. Curr. Biol. 27, R213–R214. 10.1016/j.cub.2017.02.020 28324733

[B128] Scopa C. Palagi E. (2016). Mimic me while playing! Social tolerance and rapid facial mimicry in macaques (Macaca tonkeana and Macaca fuscata). J. Comp. Psychol. 130, 153–161. 10.1037/com0000028 27078077

[B129] Shao Q. Wang H. Zhu P. Dong M. (2021). Group emotional contagion and simulation in large-scale flight delays based on the two-layer network model. Phys. A. Stat. Mech. Appl. 573:125941. 10.1016/j.physa.2021.125941

[B130] Shaobo L. Lizhong Y. Tingyong F. Jian L. (2009). Evacuation from a classroom considering the occupant density around exits. Phys. A. Stat. Mech. Appl. 388, 1921–1928. 10.1016/j.physa.2009.01.008

[B131] Sharma N. Pokharel S. S. Kohshima S. Sukumar R. (2020). Behavioural responses of free-ranging Asian elephants (*Elephas maximus*) towards dying and dead conspecifics. Primates 61, 129–138. 10.1007/s10329-019-00739-8 31428950

[B132] Simner M. L. (1971). Newborn's response to the cry of another infant. Dev Psychol. 5, 136–150. 10.1037/h0031066

[B133] Siviy S. M. Panksepp J. (1987). Sensory modulation of juvenile play in rats. Dev. Psychobiol. 20, 39–55. 10.1002/dev.420200108 3556783

[B134] Smith M. L. Hostetler C. M. Heinricher M. M. Ryabinin A. E. (2016). Social transfer of pain in mice. Sci. Adv. 2:e1600855. 10.1126/sciadv.1600855 27774512 PMC5072181

[B135] Stephen J. G. Sujeong K. Ming C. L. Dinesh M. (2011). “Simulating heterogeneous crowd behaviors using personality trait theory,” in Proceedings of the 2011 ACM SIGGRAPH/Eurographics Symposium on Computer Animation (SCA '11) (New York, NY: Association for Computing Machinery), 43–52. 10.1145/2019406.2019413

[B136] Sundman A. S. Van Poucke E. Svensson H. A. Faresjo A. Theodorsson E. Jensen P.. (2019). Long-term stress levels are synchronized in dogs and their owners. Sci. Rep. 9:7391. 10.1038/s41598-019-43851-x 31171798 PMC6554395

[B137] Tang J. Yu G. Yao X. (2021). Emotional contagion in the online depression community. Healthcare 9:1609. 10.3390/healthcare9121609 34946335 PMC8700837

[B138] Thomas S. Nam C. J. (2013). Exploring multi-stage mood contagion in a leader activation and member propagation (LAMP) model. Organ Behav. Hum. Decis. Process. 122, 127–140. 10.1016/j.obhdp.2013.06.003

[B139] Vonk J. (2019). Emotional contagion or sensitivity to behavior in ravens? Proc. Natl. Acad. Sci. U. S. A. 116, 18168. 10.1073/pnas.1909864116 31431538 PMC6744878

[B140] Waller B. M. Cherry L. (2012). Facilitating play through communication: significance of teeth exposure in the gorilla play face. Am. J. Primatol. 74, 157–164. 10.1002/ajp.21018 22512019

[B141] Wang Y. Hamilton A. F. (2013). Understanding the role of the 'self' in the social priming of mimicry. PLoS One 8:e60249. 10.1371/journal.pone.0060249 23565208 PMC3614954

[B142] Wenig K. Boucherie P. H. Bugnyar T. (2021). Early evidence for emotional play contagion in juvenile ravens. Anim. Cogn. 24, 717–729. 10.1007/s10071-020-01466-0 33420859 PMC8238721

[B143] What J. B. (2012). What makes online content viral? Strategic Direction 28, 192–206. 10.1108/sd.2012.05628haa.014

[B144] Wojczulanis-Jakubas K. Plenzler J. Jakubas D. (2019). Indications of contagious behaviours in the southern elephant seal: an observational study. Behaviour 156, 59–77. 10.1163/1568539X-00003530

[B145] Xingli L. Zhongfei G. Hua K. Xuecen B. Yanhong F. (2019). Effect of dangerous source on evacuation dynamics in pedestrian counter flow. Phys. A. Stat. Mech. Appl. 533:122047. 10.1016/j.physa.2019.122047

[B146] Yao Z. Ni Z. Zhang B. Du J. (2022). Do Informational and emotional elements differ between online psychological and physiological disease communities in China? A comparative study of depression and diabetes. Int. J. Environ. Res. Public Health 19:2167. 10.3390/ijerph19042167 35206355 PMC8872467

[B147] Zhang G. Lu D. Jia X. (2022). Emotional contagion in physical-cyber integrated networks: the phase transition perspective. IEEE Trans. Cybern. 52, 7875–7888. 10.1109/TCYB.2021.3052766 33600340

[B148] Zhang W. Song Y. Chai T. Liao G. Zhang L. Jia Q.. (2020). Lipidomics perturbations in the brain of adult zebrafish (*Danio rerio*) after exposure to chiral ibuprofen. Sci. Total Environ. 713:136565. 10.1016/j.scitotenv.2020.136565 31954244

